# Supplementary material for: Unconventional CN vacancies suppress iron-leaching in Prussian blue analogue pre-catalyst for boosted oxygen evolution catalysis
Source: Nat Commun. 2019 Jun 26;10:2799. doi: 10.1038/s41467-019-10698-9 (PMC6595008; doi:10.1038/s41467-019-10698-9)
Supplement: Supplementary file 1 — Supplementary Information [file 41467_2019_10698_MOESM1_ESM.pdf]

Supplementary Information

Unconventional CN vacancies suppress iron-leaching in Prussian blue pre-catalyst for boosted oxygen evolution catalysis

Yu et al.

Supplementary Figures

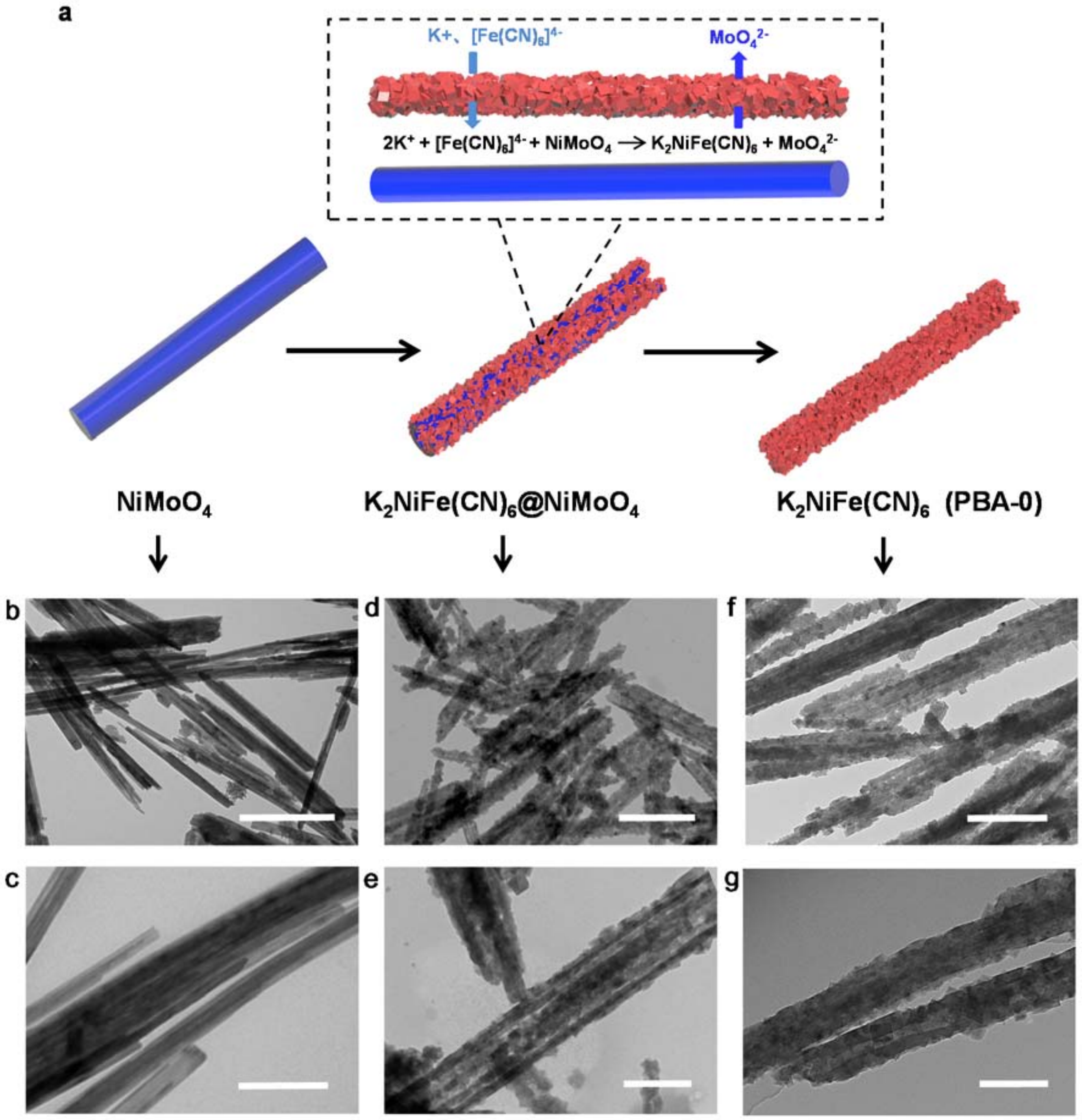

Supplementary Figure 1. Templated synthesis of PBA-0. **a,** Schematic illustration of the preparation of PBA-0 by using $NiMoO_4$ nanorods as the template. **b, c**, TEM images of $NiMoO_4$. **d, e**, TEM images of $K_2NiFe(CN)_6$@$NiMoO_4$ after 0.5 h reaction. **f, g**, TEM images of $K_2NiFe(CN)_6$ (PBA-0) after 2 h reaction. Scale bars in b, d, f: 500 nm. Scale bars in c, e, g: 200 nm. The initial $NiMoO_4$ template has the solid nanorod structures with a diameter of 50~100 nm. After the adding of $K_4Fe(CN)_6$ solution, the ion exchange of K^+ and $[Fe(CN)_6]^{4-}$ with MoO_4^{2-} was occurred to lead to the dissolution of $NiMoO_4$ template and the formation of $K_2NiFe(CN)_6$. The intermediate product of $K_2NiFe(CN)_6$@$NiMoO_4$ showed the core-shell nanorod structures (Supplementary Fig. 1d-e). After the complete reaction for 2 h, the $K_2NiFe(CN)_6$ nanorods, denoted as PBA-0, could be formed. Numerous voids were formed due to the well-known Kirkendall effect of various diffusion rates in these chemical species[1]. It was noted that the hollow nanotube structures became more visible when using the $CoMoO_4$ nanrods with a larger diameter of 150~200 nm as the template (Supplementary Fig. 29)

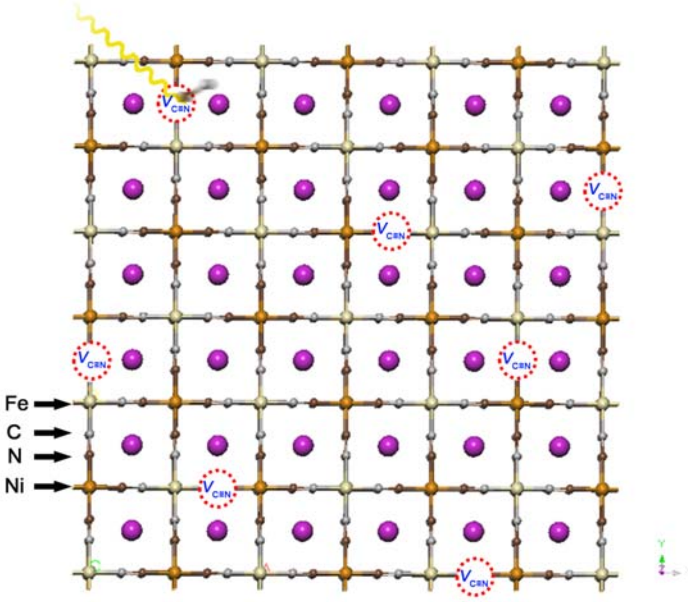

Supplementary Figure 2. Schematic illustration of V_{CN} formed in Ni-Fe PBA lattice. The generation of V_{CN} was obtained through the N_2 plasma bombardment.

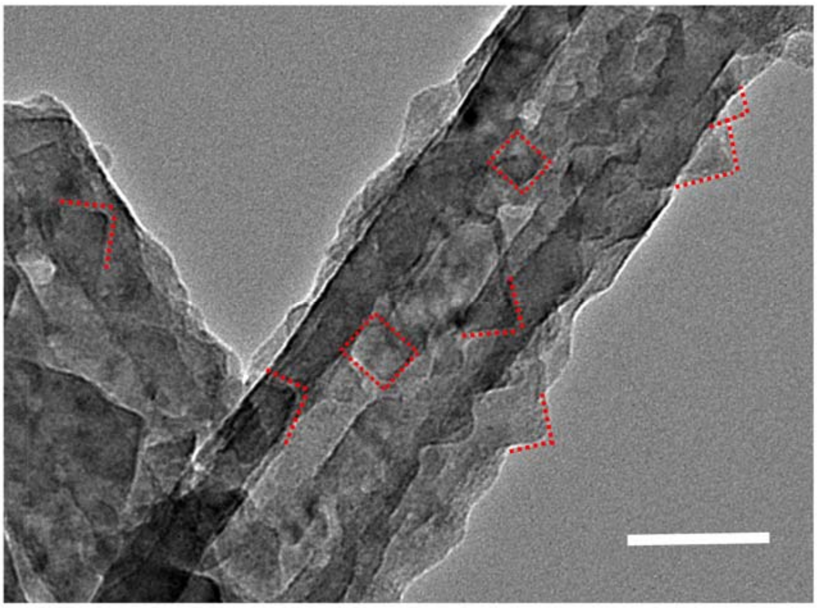

Supplementary Figure 3. TEM Characterization of PBA-60. Scale bar, 50 nm. The red dashed line showed that PBA-60 is constitutive of the small PBA nanocubes with the diameters of about 20 nm.

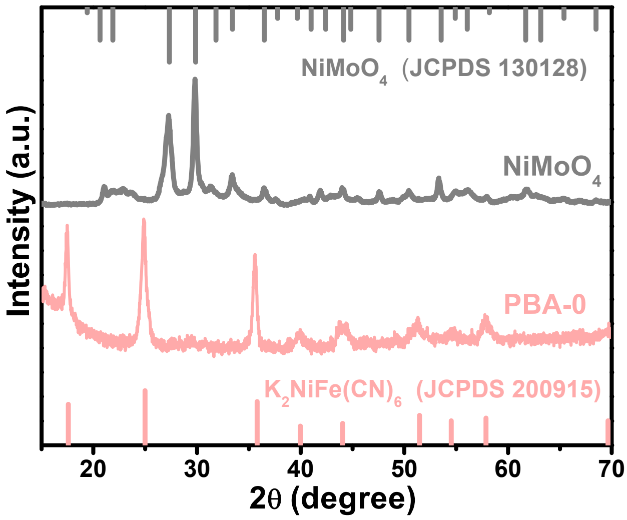

Supplementary Figure 4. XRD characterization. The XRD patterns showed that the starting $NiMoO_4$ template was totally converted into PBA-0 with the cubic $K_2NiFe(CN)_6$ phase.

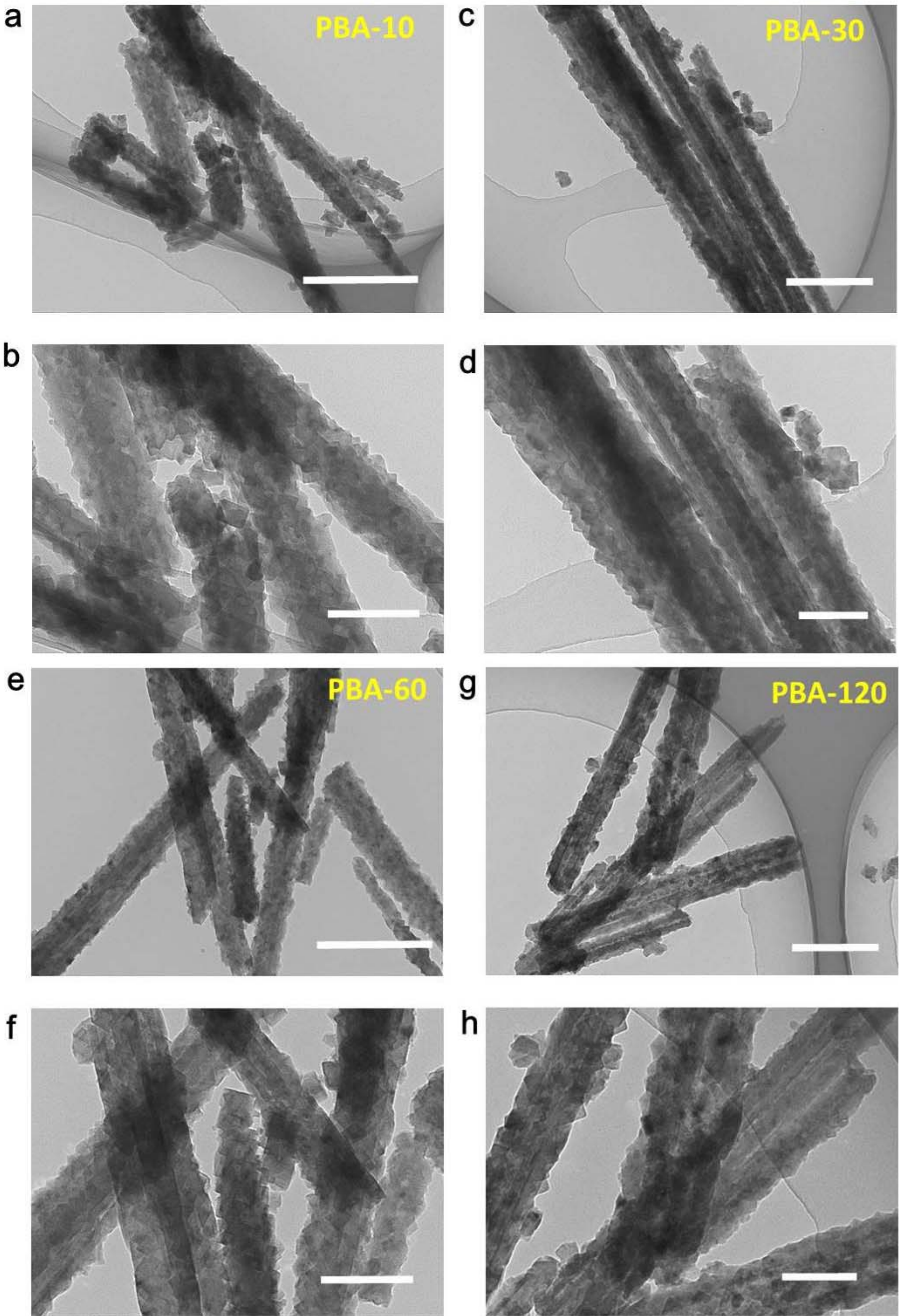

Supplementary Figure 5. TEM Characterization. a, b, PBA-10; **c, d**, PBA-30; **e, f**, PBA-60; **g, h**, PBA-120. Scale bars in a, c, e, g: 500 nm; Scale bars in b, d, f, h: 200 nm. With the plasma bombardment time increased from 10 to 120 min, the morphology almost kept unchanged, indicating the robust structure stability.

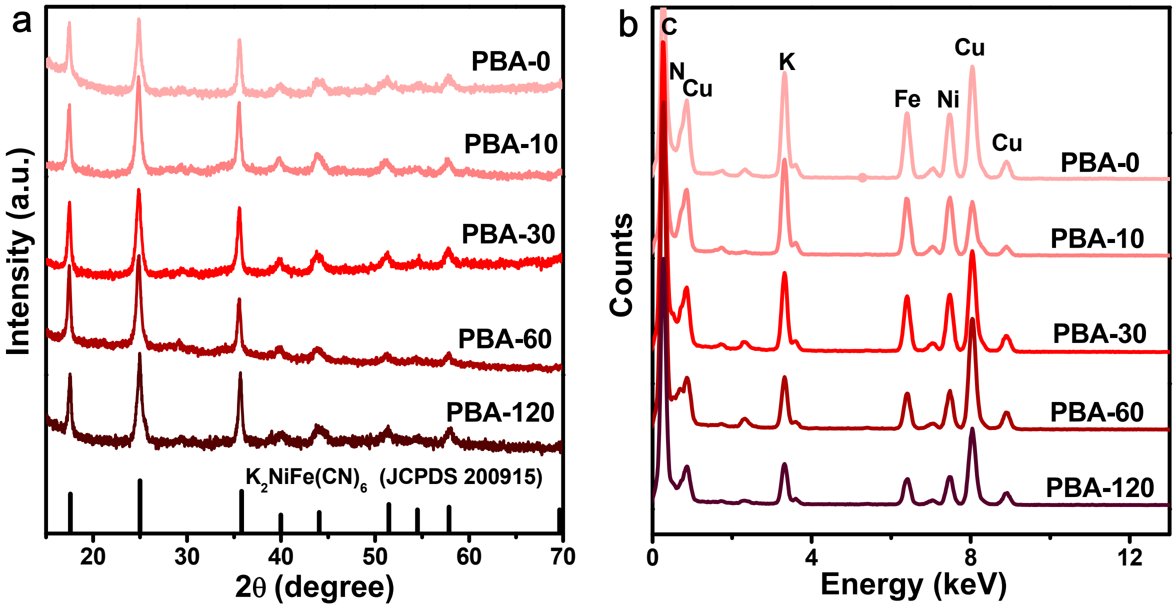

Supplementary Figure 6. XRD and EDX characterization. a, b, XRD patterns and EDX spectra of various samples. With the plasma bombardment time increased from 0 to 120 min, the XRD patterns kept unchanged, indicating that the formation of V_{CN} did not affect the crystal phase. EDX spectra of all samples showed the coexistence of K, Ni, Fe, C, and N. The Fe/Ni atomic ratios obtained from EDX for PBA-0, PBA-10, PBA-30, PBA-60, and PBA-120 were 1.06, 1.05, 1.03, 1.05, and 0.97, respectively. The decreased Fe/Ni atomic ratio for PBA-120 is mainly due to the Fe loss with the long bombardment time, which is consistent with the ICP-AES and XPS results (Supplementary Tables 4,5).

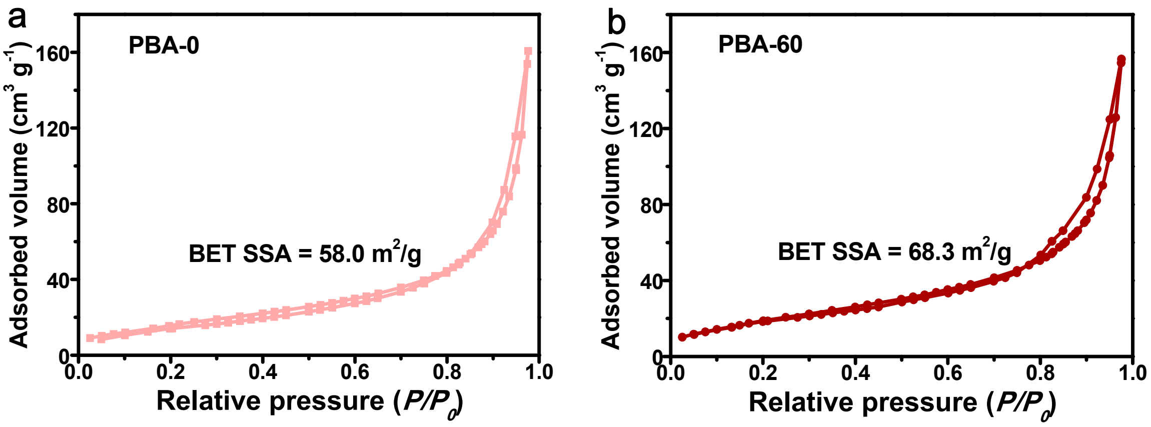

Supplementary Figure 7. Nitrogen adsorption-desorption isotherms of PBA-0 and PBA-60. Our synthesized method by using $NiMoO_4$ as the template can lead to a high mesoporous specific surface area of about 60 m^2 g^{-1} for PBA-0 and PBA-60, which is much larger than that of the previously reported solid PBA cubes (about 10 m^2 g^{-1}) *via* the conventional synthesis method[2,3]. The porous structures will benefit the access of the electrolyte and reactants to active sites to boost the catalytic activity

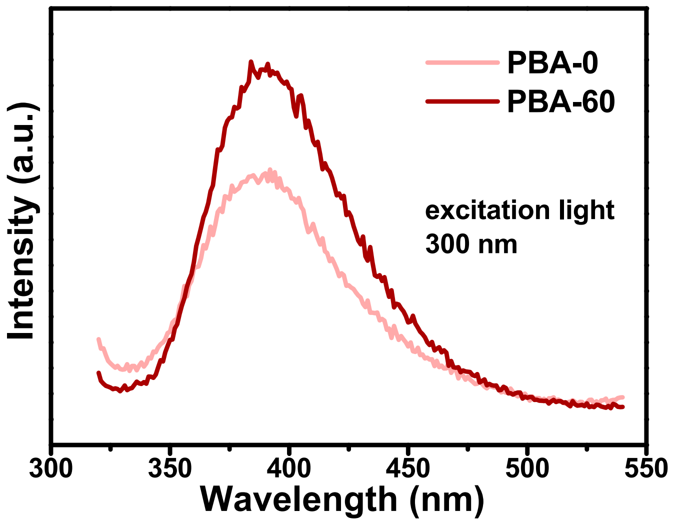

Supplementary Figure 8. PL spectra of PBA-0 and PBA-60. The excitation light is 300 nm. The tested sample mass of PBA-0 and PBA-60 is same. PBA-60 has the stronger PL signals than PBA-0, probably due to the high quantum efficiency of excitons localized at the V_{CN} sites[4,5].

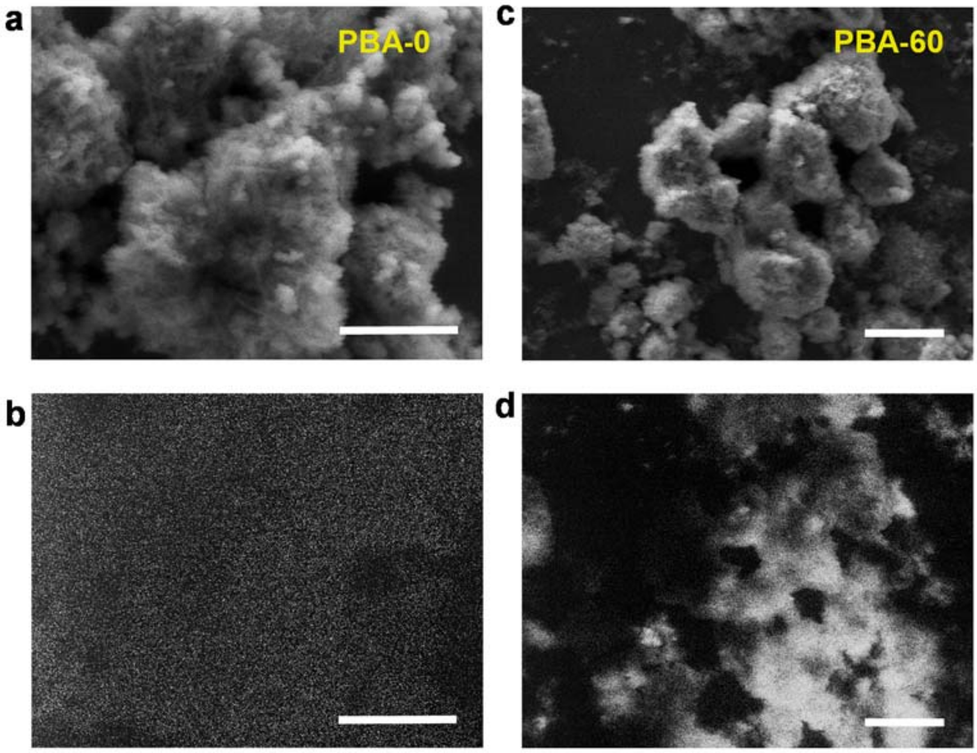

Supplementary Figure 9. CL images of PBA-0 and PBA-60. a, SEM image of PBA-0. **b,** CL image of PBA-0. Scale bars in a, b: 5 μm. **c,** SEM image of PBA-60. **d,** CL image of PBA-60. Scale bars in c, d: 10 μm. PBA-60 has the stronger CL signals than PBA-0, probably due to the high quantum efficiency of excitons localized at the V_{CN} sites [5,6].

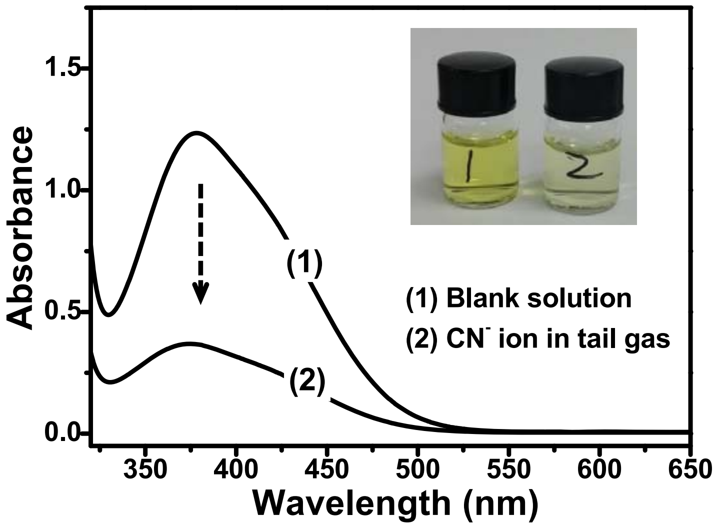

Supplementary Figure 10. The detection of cyanide in tail gas solution. The 0.4wt% ninhydrin and 2wt% Na_2CO_3 mixture aqueous solution were used to absorb cyanide in tail gas. Due to the low cyanide content in tail gas, we performed on approximately 10 batches samples to collect more cyanide. Ninhydrin was used as the color indicator to detect cyanide according to the previous literatures[7]. Ninhydrin can react with Na_2CO_3 to form the yellow product and cyanide can disassemble the product to weaken the yellow color. The recommended detection procedure was shown as followed. We bubbled the 0.4wt% ninhydrin and 2wt% Na_2CO_3 mixed solution with tail gas produced during the synthesis process. Then the mixed solution was stirred vigorously and the absorbance was measured in a spectrophotometer after 30 min. The blank solution was prepared by adding ninhydrin solution to Na_2CO_3 solution without bubbling with tail gas, which has a yellow color (sample 1 in inset). The weaker color for sample 2 showed that the tail gas solution contained a certain amount of CN^- ion.

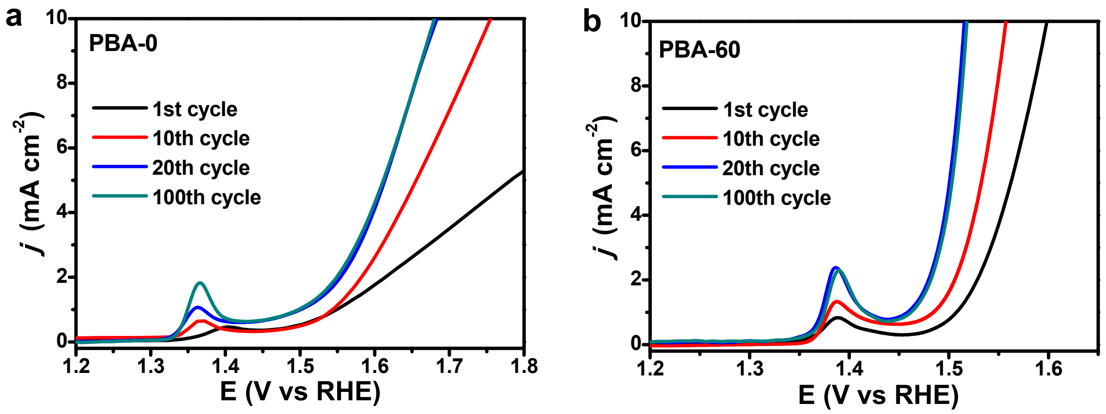

Supplementary Figure 11. OER activation process. a,b, OER polarization curves of PBA-0 and PBA-60 recorded at a sweep rate of 5 mV s^{-1} after given number of cycles between 0 and 0.8 V vs. Ag/AgCl at a sweep rate of 100 mV s^{-1}. It can be seen that the OER activity became stable after 20 CV cycles, and therefore 20 CV cycles were chosen as the activation time.

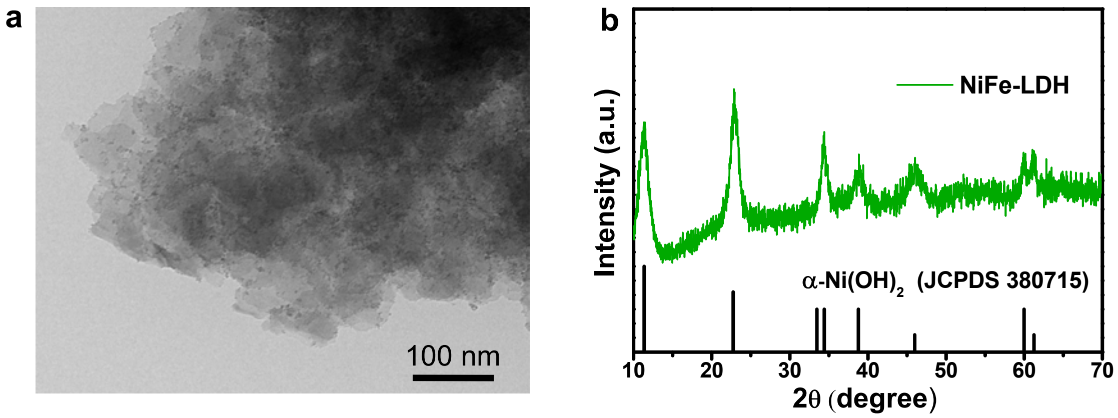

Supplementary Figure 12. The characterization of NiFe-LDH. a, TEM image of NiFe-LDH. **b,** XRD pattern of NiFe-LDH. NiFe-LDH nanoplates were synthesized in a simple solvothermal system according to Dai's work[8]. The XRD pattern of the product was consistent with the α–phase $Ni(OH)_2$.

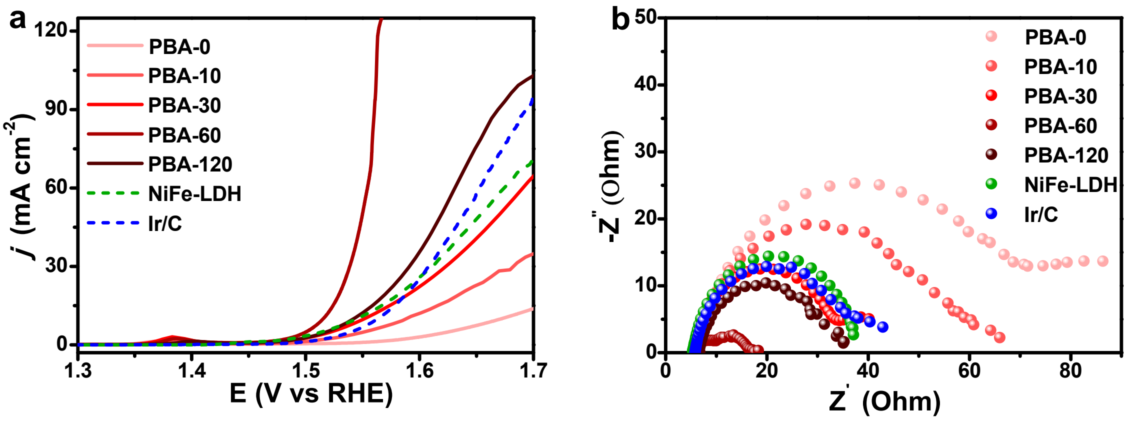

Supplementary Figure 13. OER characterization. a, Polarization curves at high current density. **b,** Nyquist plots were tested at applied overpotential of 370 mV without iR-correction. The results show that the smallest charge transfer resistance (R_{ct}) of 11 ohms was enabled on the PBA-60 catalyst, comparing favorably to other studied catalysts.

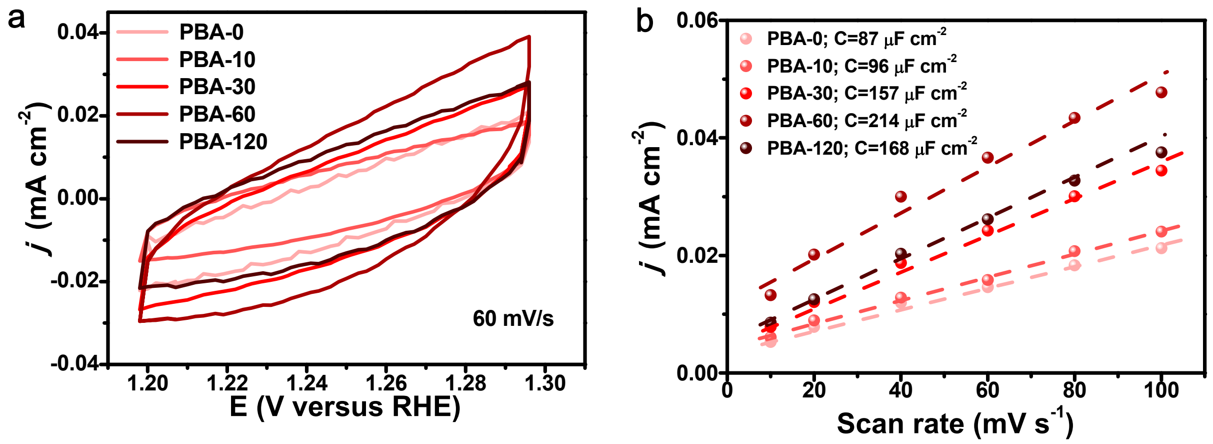

Supplementary Figure 14. The double-layer capacitance (C_{dl}) tests. a, CV curves of various catalysts within a potential of 1.2-1.3 V versus RHE. **b,** The double-layer capacitance (C_{dl}) of various catalysts. The results reveal that PBA-60 delivers the largest C_{dl} values of 214 μF cm^{-2}, much larger than that of other studied catalysts.

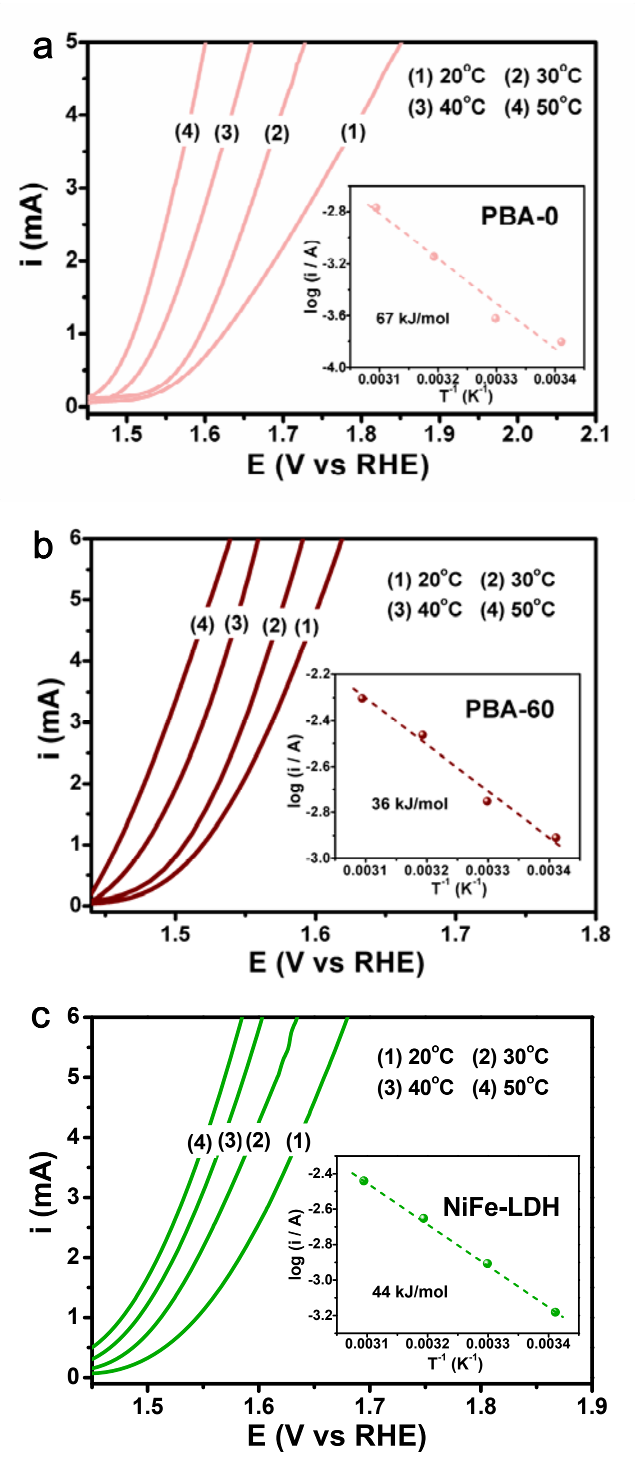

Supplementary Figure 15. The tests of the apparent electrochemical activation energy. a-c, The OER polarization of PBA-0, PBA-60, and NiFe-LDH catalysts tested at 20, 30, 40, and 50 ºC at applied overpotential of 300 mV without iR-corrected. Inset: Arrhenius plot of the inverse temperature versus the log of the current. PBA-60 has the lowest apparent activation energy value of 36 kJ mol^{-1}.

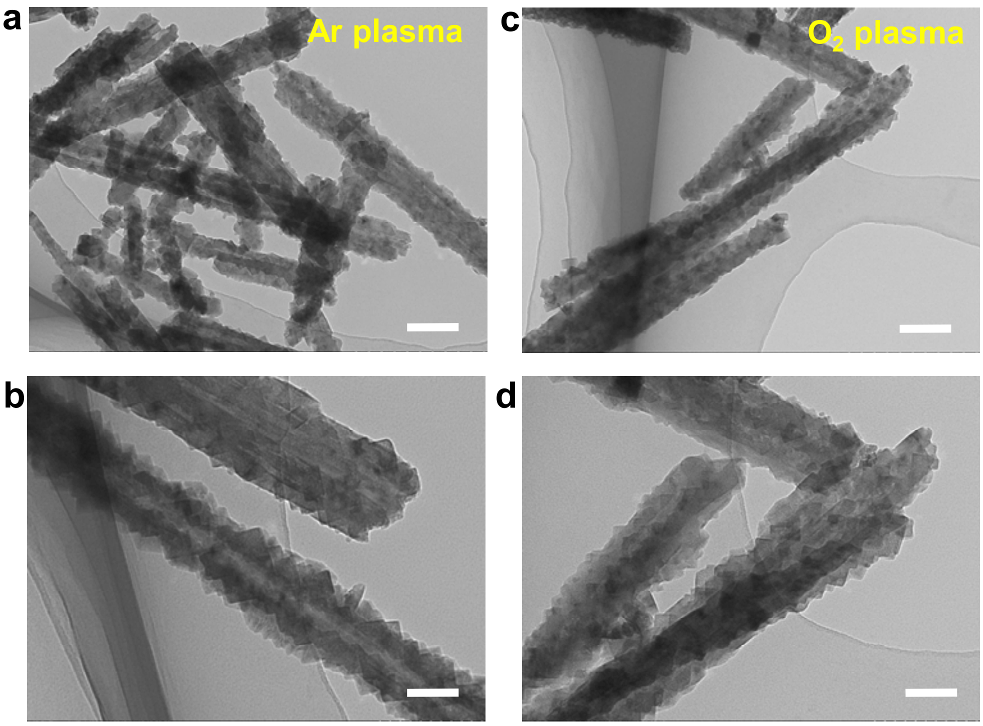

Supplementary Figure 16. TEM Characterization of samples treated in various plasma atmospheres. a, b, TEM images of PBA-60 obtained by Ar plasma bombardment. **c, d**, TEM images of the sample of PBA-60 obtained by O_2 plasma bombardment. Scale bars in a, c: 200 nm; Scale bars in b, d: 100 nm. Adjusting the plasma bombardment atmospheres, the morphology almost kept unchanged, indicating that the plasma atmospheres have little effect on the morphology.

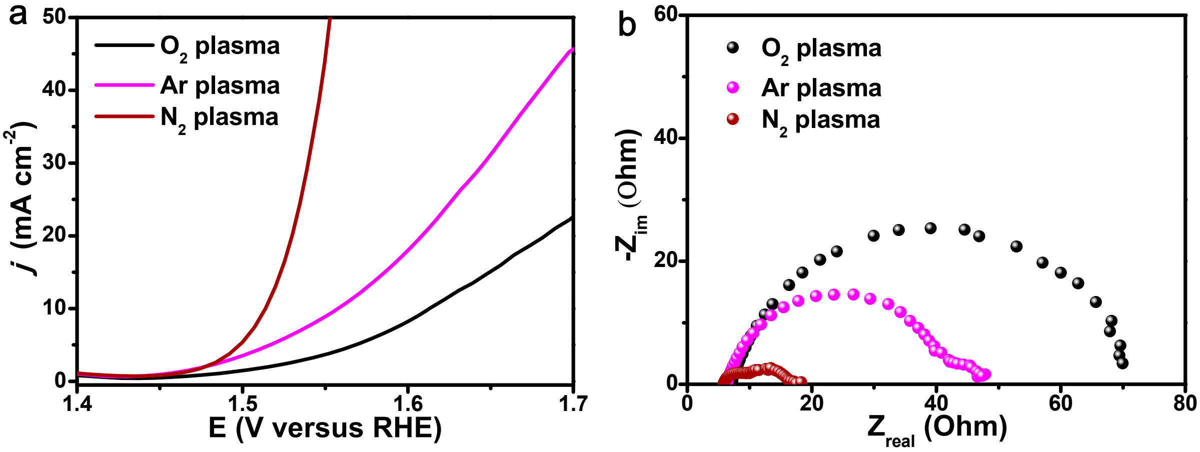

Supplementary Figure 17. The effect of plasma bombardment atmospheres on OER activity. a, b, Polarization curves and EIS Nyquist plots of PBA-60 obtained by O_2, Ar, or N_2 plasma bombardment. The result showed that PBA-60 obtained by N_2 plasma bombardment has the optimal OER activity and the fastest Faradaic charge transfer.

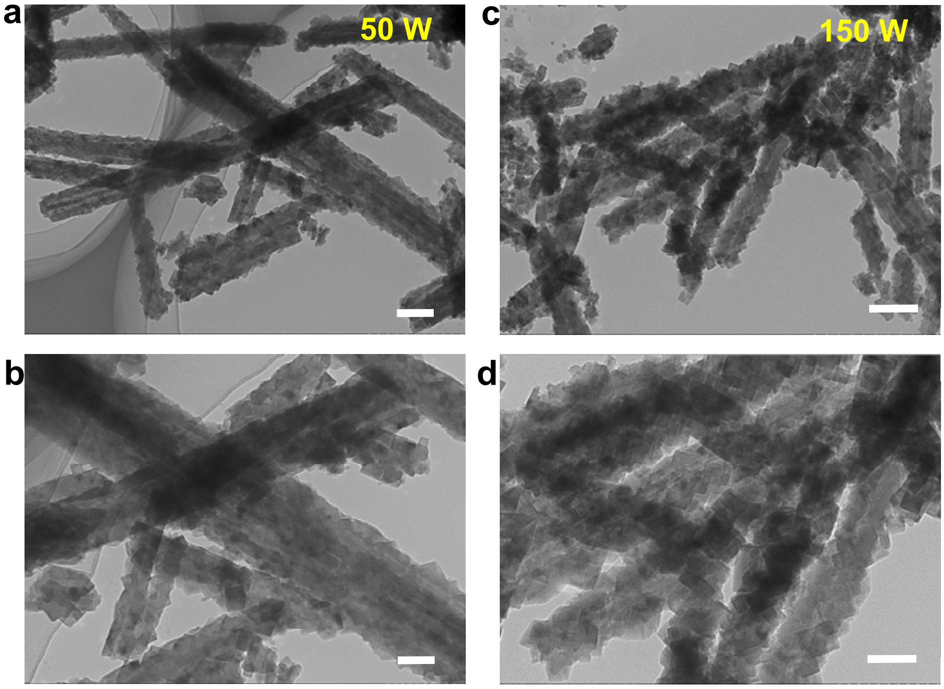

Supplementary Figure 18. TEM Characterization of samples treated at various plasma bombardment power. a, b, TEM images of PBA-60 obtained by N_2 plasma bombardment at a power of 50 W. **c, d**, TEM images of PBA-60 obtained by N_2 plasma bombardment at a power of 150 W. Scale bars in a, c: 200 nm; Scale bars in b, d: 100 nm. Adjusting the plasma bombardment power, the morphology almost kept unchanged, indicating that the plasma powers have little effect on the morphology.

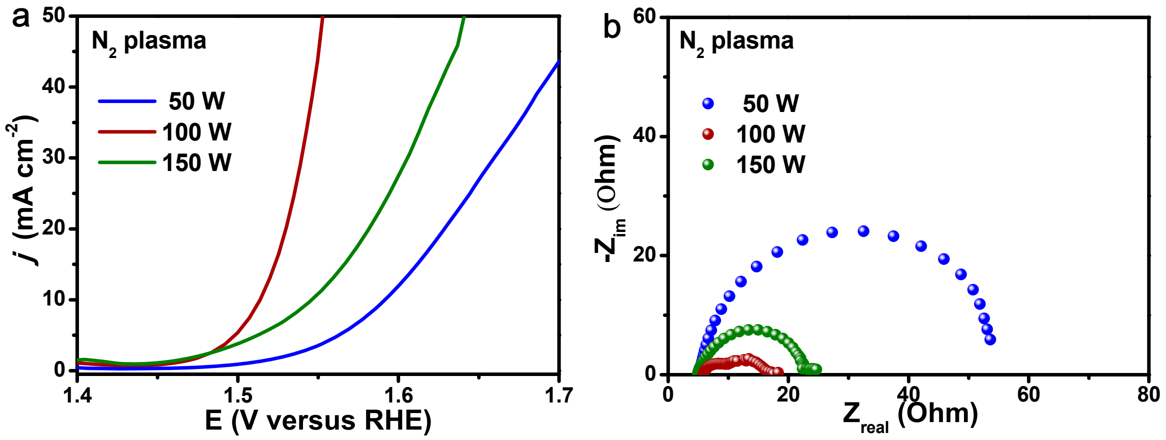

Supplementary Figure 19. The effect of N_2 plasma power on OER activity. a, b, Polarization curves and Nyquist plots of PBA-60 obtained by N_2 plasma bombardment at various powers. The result showed that the catalyst treated by 100 W N_2 plasma has the optimal OER activity and the fastest Faradaic charge transfer. The worse OER performance for catalyst treated by 50 W could be attributed to the insufficient V_{CN}. The worse OER performance for catalyst treated by 150 W could be attributed to the loss of NiFe-based active sites with the large plasma power.

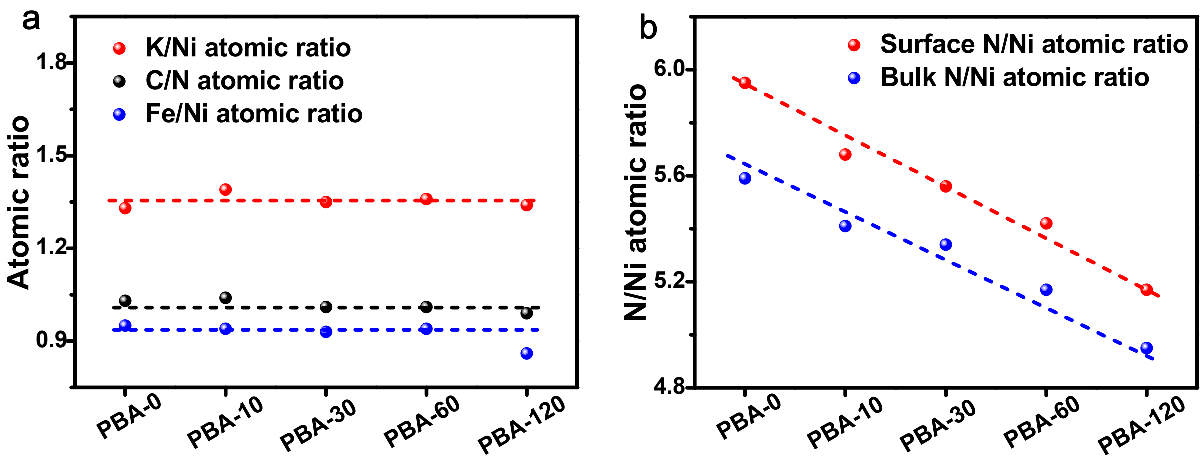

Supplementary Figure 20. Elemental atomic ratios. a, Bulk K/Ni, C/N, Fe/Ni atomic ratios (Supplementary Table 4). **b,** Bulk and surface N/Ni atomic ratios (Supplementary Tables 4,5). We found that both bulk and surface atomic ratios of K/Ni, Fe/Ni and C/N remain unchanged even bombarding for 60 minutes, but the atomic ratios of N/Ni and C/Fe decreased with time. This unambiguously suggests the formation of sole V_{CN} in PBA without any other vacancy defects. However, when we bombarded the PBA for 120 minutes, we observed a decreased Fe/Ni atomic ratio, indicative of some Fe vacancies formed in the structure. Since FeC_6 and NiN_6 octahedra are bridged through CN ligand, the decreased C/Fe or N/Ni atomic ratio can reflect the V_{CN} concentration. However, due to the large deviation of the C content derived from XPS, the surface C/Fe atomic ratios of various samples are not accurate. Therefore, we used the N/Ni atomic ratio rather than the C/Fe atomic ratio to determine the V_{CN} contents. The bulk N/Ni atomic ratios of PBA-0, PBA-10, PBA-30, PBA-60, and PBA-120 were 5.59, 5.41, 5.34, 5.17, and 4.95, respectively. Therefore, we can obtain the bulk V_{CN} contents of PBA-10, PBA-30, PBA-60, and PBA-120 samples are ~3.2%, 4.5%, 7.5%, and 11.4%, respectively, which are slightly lower than those corresponding surface V_{CN} contents (Fig. 4a and Supplementary Tables 4,5).

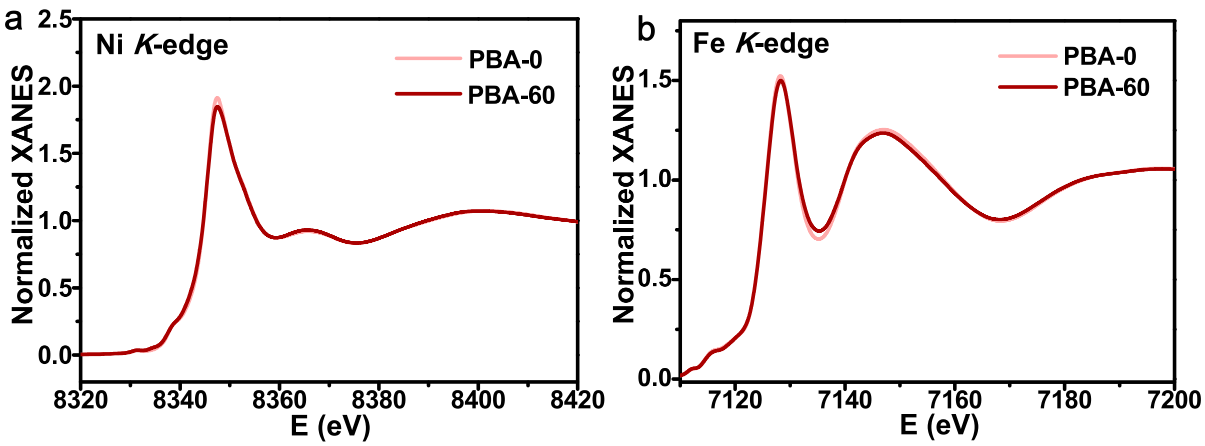

Supplementary Figure 21. XANES curves of PBA-0 and PBA-60. a, The Ni K-edge XANES spectra of PBA-0 and PBA-60. **b,** The Fe K-edge XANES spectra of PBA-0 and PBA-60. PBA-0 and PBA-60 have the nearly identical XANES spectra for both Ni and Fe K-edge, indicating the similar chemical states in the bulk.

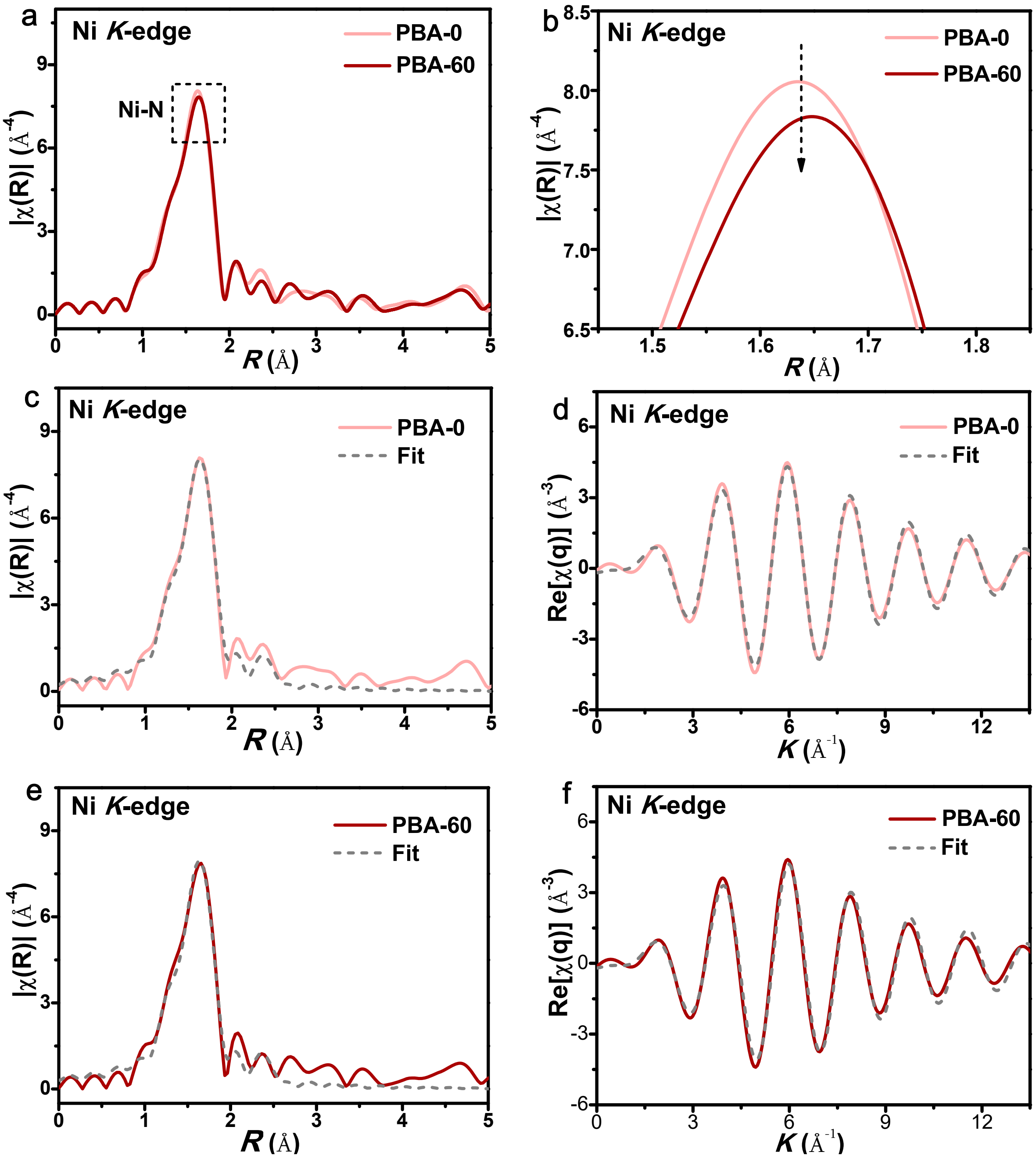

Supplementary Figure 22. EXAFS curves of Ni K-edge. a, b, The Ni K-edge EXAFS curves of PBA-0 and PBA-60. **c, d,** The raw and fitting Ni K-edge EXAFS curves of PBA-0. **e, f,** The raw and fitting Ni K-edge EXAFS curves of PBA-60. The fitting results (Supplementary Table 6) showed that the coordination numbers of Ni-N within PBA-0 and PBA-60 are 5.9 and 5.5, respectively. The decrease Ni-N coordination number is due to the formation of V_{CN}, implying that Ni atom became the coordinatively unsaturated metal sites after the plasma bombardment.

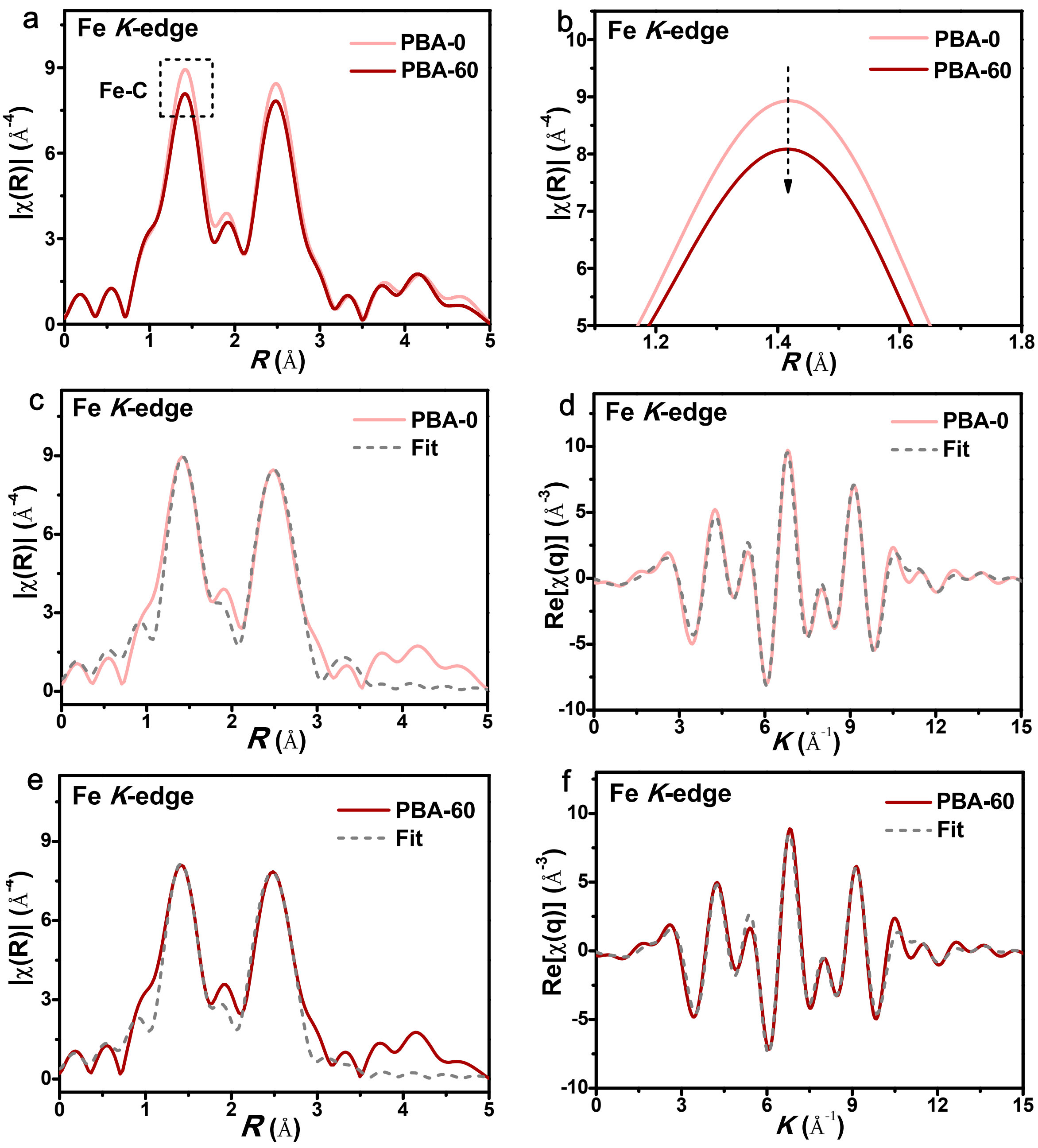

Supplementary Figure 23. EXAFS curves of Fe K-edge. a, b, The Fe K-edge EXAFS curves of PBA-0 and PBA-60. **c, d,** The raw and fitting Fe K-edge EXAFS curves of PBA-0. **e, f,** The raw and fitting Fe K-edge EXAFS curves of PBA-60. The first peak at about 1.4 Å for Fe K-edge spectra could be attributed to the Fe-C bond[9-11]. The fitting results (Supplementary Table 6) showed that the coordination numbers of Fe-C within PBA-0 and PBA-60 are 6.1 and 5.7, respectively. The decrease Fe-C coordination number is due to the formation of V_{CN}, implying that Fe atom became the coordinatively unsaturated metal sites after the plasma bombardment. The second EXAFS peak at about 2.5 Å for Fe K-edge spectra could be attributed to the Fe-N and Fe-Fe bond, which is also observed in the previously reported work[9-11].

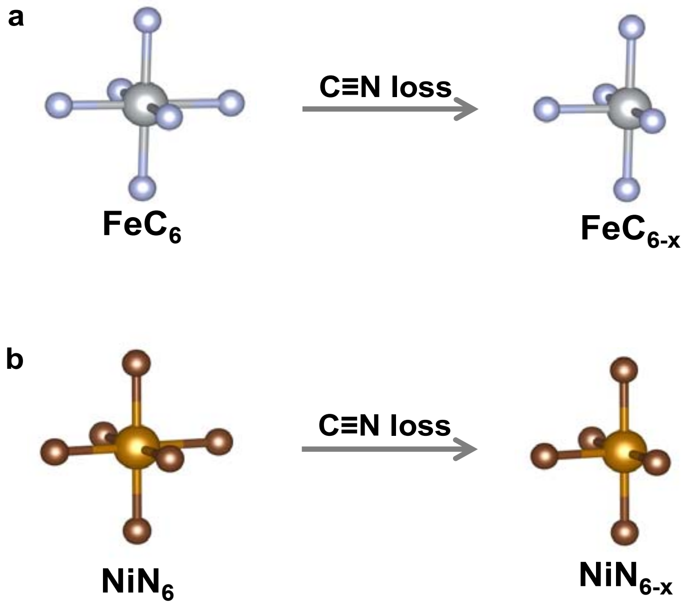

Supplementary Figure 24. Schematic illustration of the decreased coordination number after the generated V_{CN}. Since FeC_6 and NiN_6 octahedra are bridged through CN ligand, the decreased Fe-C and Ni-N coordination numbers of PBA-60 in comparison with PBA-0 are due to the generated V_{CN} after plasma bombardment (Supplementary Table 6).

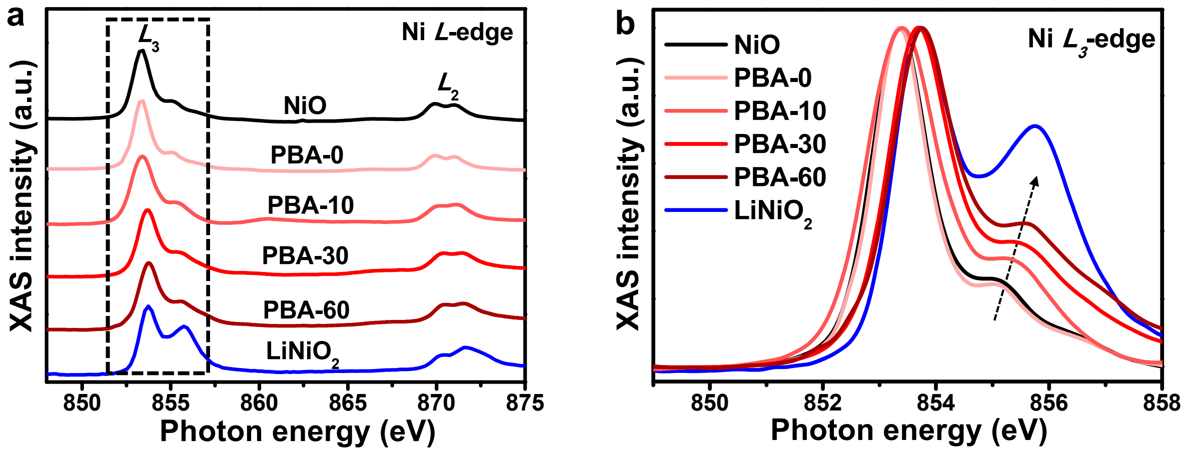

Supplementary Figure 25. XAS curves of Ni L-edges. a, The Ni L-edge spectra of various samples. **b**, The magnified Ni L_3-edges of various samples. NiO (Ni^{2+}) reference was purchased from Sigma-Aldrich#without any further purification. $LiNiO_2$ was chosen as the Ni^{3+} reference sample because it contains Ni^{3+} (ref.[12,13]), and it was synthesized by a solid state method according to Tong's work[14]. In the magnified Ni L_3-edges, $LiNiO_2$ has the stronger shoulder peak (at 855~856 eV) than NiO, suggesting the increasing Ni^{3+} component[12,13]. Therefore, the increased peak intensity from PBA-0 to PBA-60 indicated that surface Ni^{2+} ions were partly oxidized to Ni^{3+} after N_2 plasma bombardment.

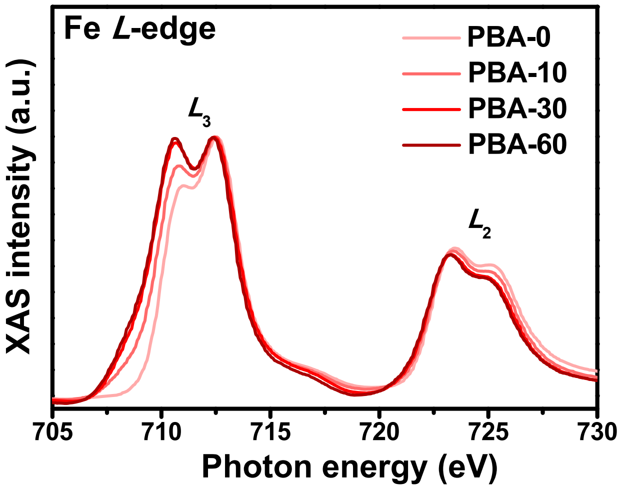

Supplementary Figure 26. XAS curves of Fe L-edges. The magnified Fe L_3-edges were shown in Fig. 4e.

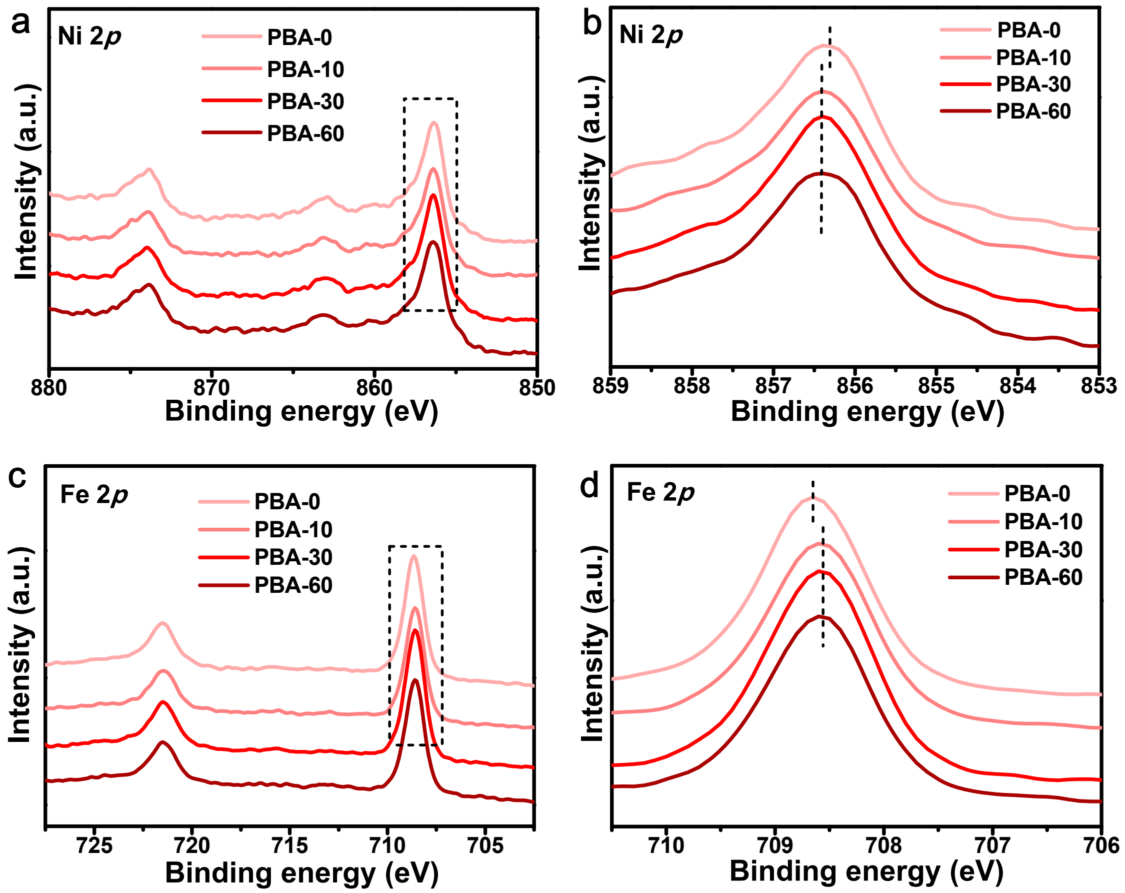

Supplementary Figure 27. XPS analysis. a, b, Ni 2*p* XPS spectra of various samples. **c, d,** Fe 2*p* XPS spectra of various samples. After the plasma bombardment, the Ni and Fe peaks shifted to higher and lower binding energy, respectively, indicating a partial electron transfer between them. The surface oxidation states of Ni and Fe is increased and decreased, respectively, which is consistent with the XAS results.

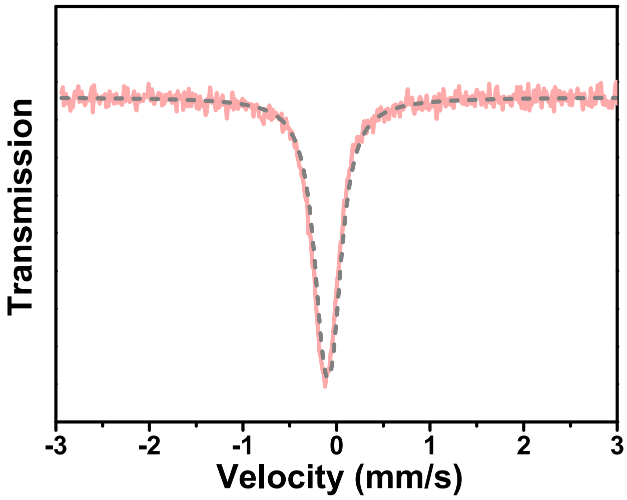

Supplementary Figure 28. Mössbauer spectrum collected at room temperature of PBA-0. The Mössbauer spectrum of PBA-0 showed a singlet absorption peak, which corresponds to the low-spin Fe^{2+} species[2,15]. Because Mössbauer spectrum is the bulk characterization technique, the bulk oxidation state of Fe in our NiFe PBAs is +2, consistent with the previous work[2,11]. The weak peak of Fe^{3+} species detected in Raman spectra (Fig. 4c) is due to the partial surface Fe^{2+} readily oxidized to Fe^{3+} in the lab environment[2,16], which returns to its original state after the V_{CN} formed (Fig. 4c,e, Supplementary Figs. 26-27).

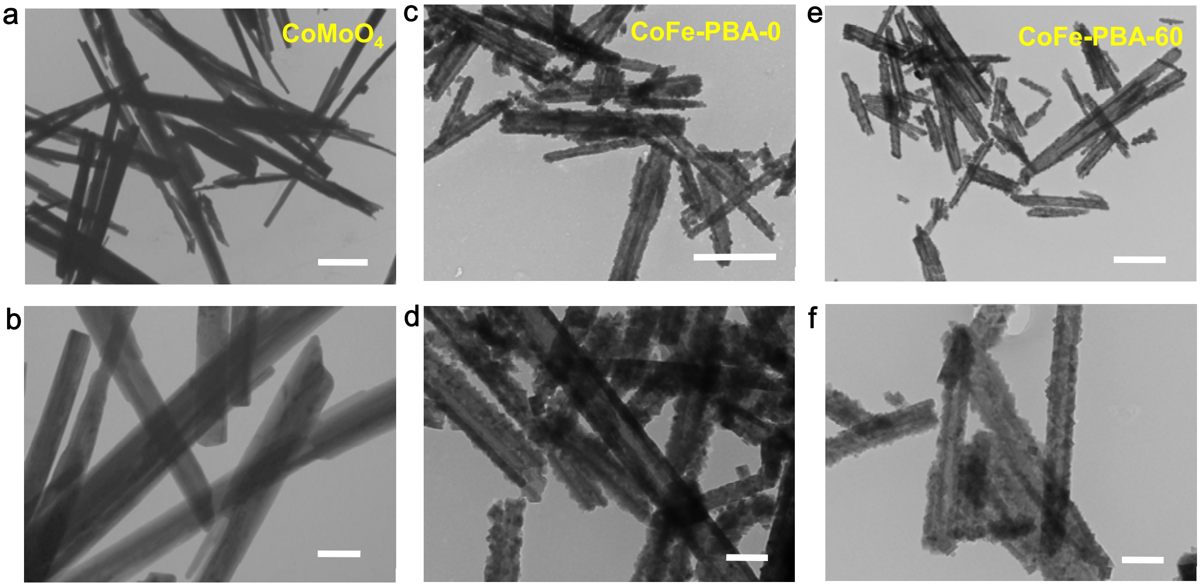

Supplementary Figure 29. Characterization of CoFe-PBA-60 derived from $CoMoO_4$. a, b, TEM images of $CoMoO_4$ nanorods. **c, d**, TEM images of CoFe-PBA-0. **e, f**, TEM images of CoFe-PBA-60. Scale bars in a, c, e: 1 µm. Scale bars in b, d, f: 200 nm. The similar synthesis process by using $CoMoO_4$ nanorods as the template can lead to the porous CoFe-based PBA nanorods (CoFe-PBA-0). The formation of hollow CoFe-PBA-0 nanotubes could be attributed to the well-known Kirkendall effect. After the N_2 plasma bombardment of CoFe-PBA-0 for 60 min, the resultant CoFe-PBA-60 had the unchanged morphology, indicating the robust structure stability. The result showed that it is a general route to synthesize porous PBA materials through using metal molybdate as the precursor.

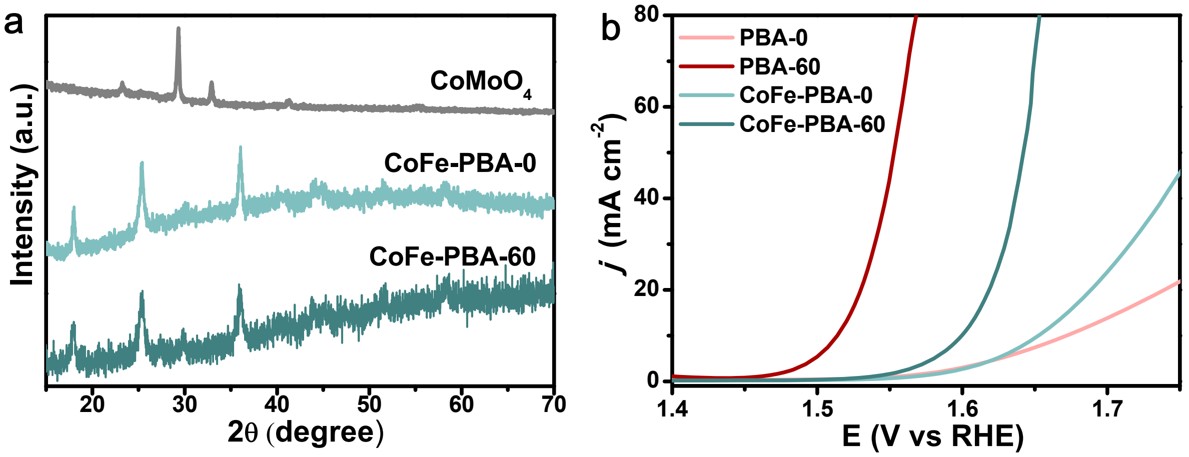

Supplementary Figure 30. Characterization of CoFe-PBA-60 derived from $CoMoO_4$. a, XRD patterns of various samples. **b**, OER polarization curves of various samples. The XRD patterns showed that the starting $CoMoO_4$ template was totally converted into CoFe-PBA-0 with the cubic $K_2CoFe(CN)_6$ phase (JCPDS 750038), which shows the similar crystal structures with PBA-0 (Supplementary Fig. 4). The same XRD patterns between CoFe-PBA-0 and CoFe-PBA-60 reveal that the plasma bombardment did not affect the crystal phase. OER polarization curves showed that it needs overpotentials of about 440, 283, 422, and 369 mV for PBA-0, PBA-60, CoFe-PBA-0, and CoFe-PBA-60 to achieve a current density of 10 mA cm^{-2}, respectively. Both PBA-60 and CoFe-PBA-60 had a better OER activity than the corresponding plasma-untreated counterparts, which could be attributed to the formation of V_{CN}. The result implies that the plasma bombardment may be a general route to enhance the OER activity of PBA-based materials. In addition, although CoFe-PBA-0 has the similar OER activity with PBA-0, the OER activity of CoFe-PBA-60 is much inferior to PBA-60, likely due to the low OER activity of Co-Fe based sites in comparison with Ni-Fe based sites[17]. It can further confirm the synergistic interplay between the unsaturated Ni and Fe sites in PBA-60 to boost its high OER activity.

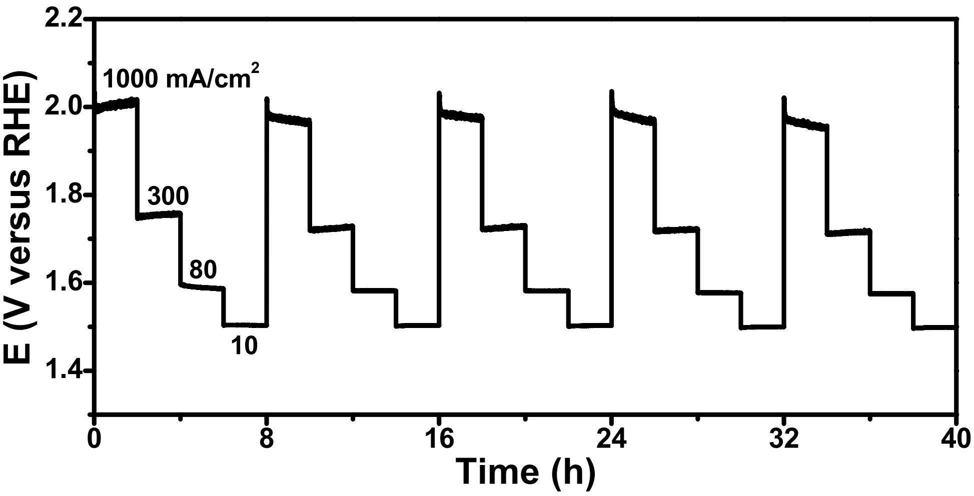

Supplementary Figure 31. The stepped chronopotentiometric curve recorded on PBA-60. The continuous current densities are 1000, 300, 80, and 10 mA cm^{-2} with each step for 2 h. The current density started at 1000 mA cm^{-2}. OER activity of PBA-60 is very stable even sustaining a current density of as high as 1,000 mA cm^{-2}, indicating the robust OER stability.

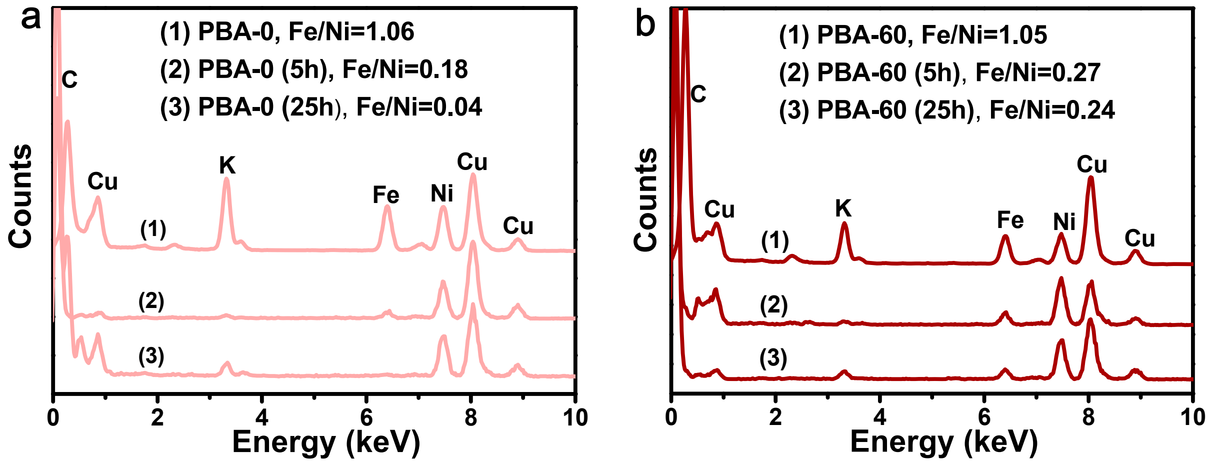

Supplementary Figure 32. EDX spectra after OER stability tests. a, EDX spectra of PBA-0 before and after 5 h and 25 h OER stability tests. **b,** EDX spectra of PBA-60 before and after 5 h and 25 h OER stability tests. PBA-60 showed the partial dissolution of Fe species at the first 5 h and after that the Fe/Ni ratio kept stable (about 0.25). However, almost all Fe species of PBA-0 was leached into the electrolyte after 25 h OER stability tests. The results showed that V_{CN}–rich PBA-60 can suppress the loss of Fe species and maintain the Ni-Fe based active sites.

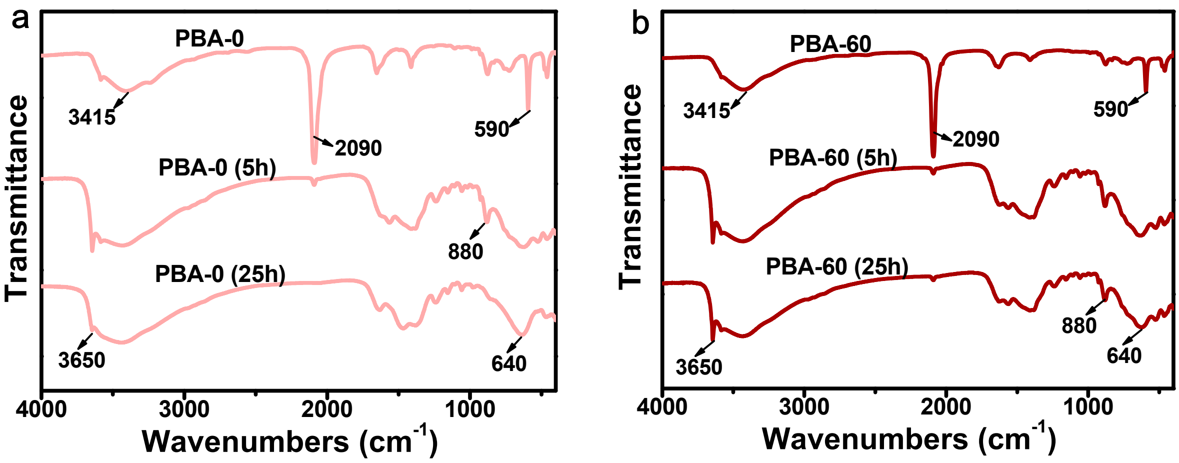

Supplementary Figure 33. FT-IR spectra after OER stability tests. a, FT-IR spectra of PBA-0 before and after 5 h and 25 h OER stability tests. **b,** FT-IR spectra of PBA-60 before and after 5 h and 25 h OER stability tests. The initial PBA-0 and PBA-60 have the similar FT-IR curves. The broad band at 3415 cm^{-1} is corresponded to the O-H stretching mode of the water molecule[18,19]. The sharp bands at 2090 and 590 cm^{-1} are the characteristic peaks of C≡N and Fe-C stretching modes, respectively[3,20,21]. After 5 h or 25 h OER stability tests, both C≡N and Fe-C stretching bands disappeared, indicating the dissolution of CN group during the OER cycling process. PBA-60 cycled for 25 h exhibit that three bands at 3650 cm^{-1}, 880 cm^{-1} and 640 cm^{-1} become dominant with time, which belongs to the O-H stretching mode in brucite-like $Ni(OH)_2$ structure[18,19], as well as the Fe-O-H (ref.[22]) and Ni-O-H bending modes[23,24], respectively, indicating the formation of Ni-Fe oxy(hydroxide) active surface layer during OER. We note that the Fe-O-H bond at 880 cm^{-1} was not detected for original PBA-0 cycled for 25 hours because of the loss of Fe, agreeing with the above EDX results.

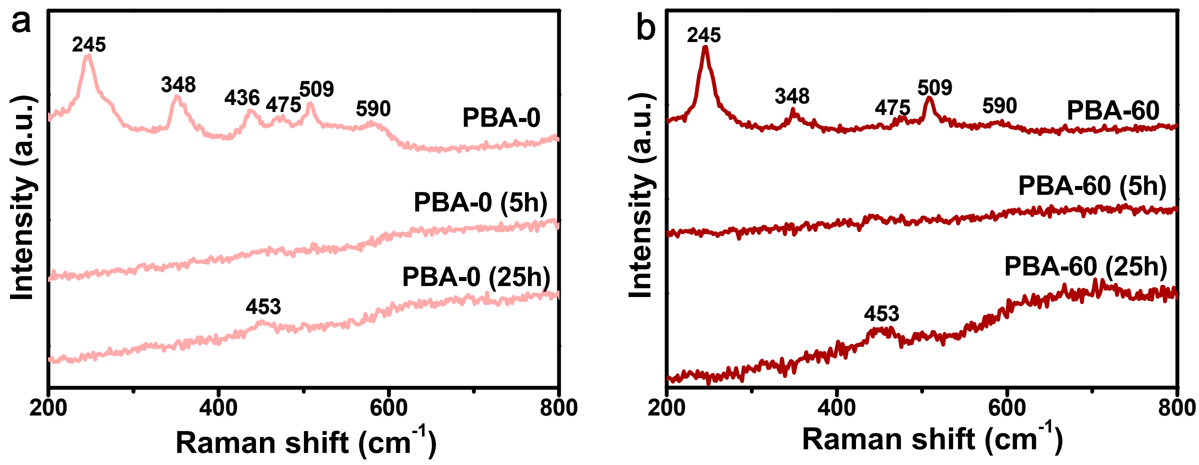

Supplementary Figure 34. Raman spectra after OER stability tests. a, Raman spectra of PBA-0 before and after 5 h and 25 h OER stability tests. **b,** Raman spectra of PBA-60 before and after 5 h and 25 h OER stability tests. A series of bands at 200~600 cm^{-1} are corresponded to the stretching or bending vibrations of metal-carbon and metal-nitrogen bonds[25-27]. The bands at 590 and 509 cm^{-1} belong to the Fe-C stretching vibrations. The bands at 475 and 436 cm^{-1} belong to the Fe-CN bending vibrations. The bands at 348 and 245 cm^{-1} belong to the Ni-N stretching vibration or Ni-NC bending vibration. After 5 h or 25 h OER cycling tests, these bands disappeared, whereas a weak band at about 453 cm^{-1} appeared, which is corresponded to the Ni-O stretching vibration.

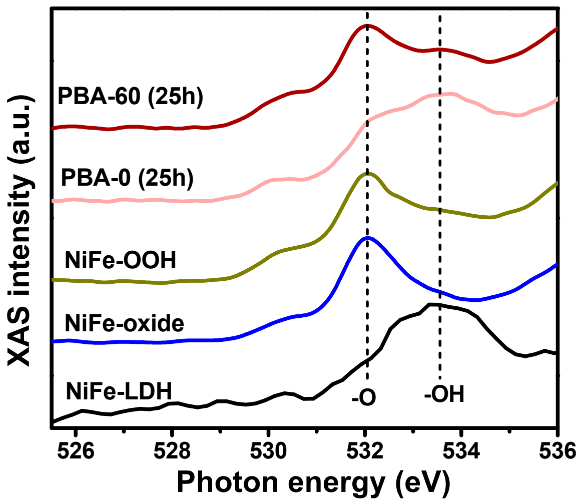

Supplementary Figure 35. XAS spectra of O K-edges. NiFe-LDH was synthesized according to Dai's work[8] (Supplementary Fig. 12). NiFe-oxide was obtained by annealing NiFe-LDH at 350 ºC in air atmosphere. NiFe-OOH was obtained by the treatment of NiFe-LDH at 1.8 V vs. RHE for 10 h under OER condition[28], which exhibited the obvious feature arising from –O and -OH species in O K-edge spectra. Meanwhile, the samples of PBA-0 and PBA-60 after OER tests showed the similar O K-edge XAS spectra with NiFe-OOH sample, indicating that the oxy(hydroxide) active layer was formed on the surface of PBA-0 and PBA-60 during OER process.

Supplementary Table 1. Positron lifetime parameters of PBA-0 and PBA-60.

Sample	τ_1 (ps)	τ_2 (ps)	τ_3 (ns)	I_1 (%)	I_2 (%)	I_3 (%)
PBA-0	250	361	2.49	44.2	55.2	0.6
PBA-60	267	388	3.25	59.4	39.5	1.1

τ_1-τ_3 represent the various positron lifetimes and I_1-I_3 are the corresponding relative intensities. The two longer components (τ_2 and τ_3) come from the large voids and the interface in the materials[29]. The measured shorter component (τ_1) of 267 ps for PBA-60 matches well with the calculated positron lifetime of 270 ps for V_{CN}, implying that positron annihilation traps at such vacancies (Supplementary Tables 1,2).

Supplementary Table 2. Calculated positron lifetime values of PBA.

Vacancy	Perfect	$V_{C/N/Ni/Fe}$	V_K	V_{FeC}	V_{NiN}	V_{CN}	V_{NiNC}	V_{FeCN}
Lifetime (ps)	249	250~252	370	255	256	270	298	299

Supplementary Table 3. Comparison of the OER activity of PBA-60 with other non-noble-metal OER catalysts with cation or/and anion vacancies in alkaline media.

Catalyst	Loading ($mg\ cm^{-2}$)	Electrolyte	Onset η (mV) at 1 $mA\ cm^{-2}$	η (mV) at 10 $mA\ cm^{-2}$	Tafel slope ($mV\ dec^{-1}$)	Ref.
$Ca_{0.9}Yb_{0.1}MnO_3$ (V_O)	N/A	0.1M KOH	385	460	68	30
$Ca_2Mn_2O_5$ (V_O)	0.255	0.1M KOH	~340	~490	149	31
CoO (V_O)	0.19	1M KOH	~275	330	44	32
Co_3O_4 (V_O)	0.136	1M KOH	~315	~400	72	33
CoO_x (V_O)	0.5	1M KOH	240	306	67	34
$CoFe_2O_4$ (V_O)	0.028	1M KOH	280	320	48	35
$Sr_2Fe_{1.3}Ni_{0.2}Mo_{0.5}O_6$ (V_O)	0.25	0.1M KOH	~300	360	59	36
$FeCoO_x$ (V_O)	0.36	0.1M KOH	~230	308	36.8	37
$CoSe_2$ (V_{Co})	0.142	0.1M KOH	260	320	44	38
$Co_{1-x}S$/rGO (V_{Co})	0.68	1M KOH	260	310	79	39
$La_{0.95}FeO_3$ (V_{La}, V_O)	0.232	0.1M KOH	365	410	48	40
CoFe-LDH (V_{Co}, V_{Fe}, V_O)	0.204	1M KOH	~255	290	36	41
NiFe-PBA (V_{CN})	0.255	1M KOH	225	283	54	This work

Supplementary Table 4. The bulk atomic ratio obtained from ICP-AES (measuring K, Fe, and Ni) and elemental analysis (measuring C and N).

Sample	K/Ni	Fe/Ni	C/N	C/Fe	N/Ni	V_{CN} content
PBA-0	1.33	0.95	1.03	6.06	5.59	0%
PBA-10	1.39	0.94	1.04	5.99	5.41	3.2%
PBA-30	1.35	0.93	1.01	5.80	5.34	4.5%
PBA-60	1.36	0.94	1.01	5.56	5.17	7.5%
PBA-120	1.34	0.86	0.99	5.70	4.95	11.4%

We found that both bulk and surface atomic ratios of K/Ni, Fe/Ni and C/N remain unchanged even bombarding for 60 minutes, but the atomic ratios of N/Ni and C/Fe decreased with time. This unambiguously suggests the formation of sole V_{CN} in PBA without any other vacancy defects. However, when we bombarded the PBA for 120 minutes, we observed a decreased Fe/Ni atomic ratio, indicative of some Fe vacancies formed in the structure.

Since FeC_6 and NiN_6 octahedra are bridged through CN ligand, the decreased C/Fe or N/Ni atomic ratio can reflect the V_{CN} concentration. However, due to the large deviation of the C content derived from XPS, the surface C/Fe atomic ratios of various samples are not accurate. Therefore, we used the N/Ni atomic ratio rather than the C/Fe atomic ratio to determine the V_{CN} contents. The bulk N/Ni atomic ratios of PBA-0, PBA-10, PBA-30, PBA-60, and PBA-120 were 5.59, 5.41, 5.34, 5.17, and 4.95, respectively. Therefore, we can obtain the bulk V_{CN} contents of PBA-10, PBA-30, PBA-60, and PBA-120 samples are ~3.2%, 4.5%, 7.5%, and 11.4%, respectively, which are slightly lower than those corresponding surface V_{CN} contents (Fig. 4a and Supplementary Tables 4,5).

Supplementary Table 5. The surface atomic ratio for different samples obtained from XPS.

Sample	K/Ni	Fe/Ni	N/Ni	V_{CN} content
PBA-0	1.65	1.06	5.95	0%
PBA-10	1.58	1.0	5.68	4.5%
PBA-30	1.66	1.02	5.56	6.6%
PBA-60	1.59	1.01	5.42	8.9%
PBA-120	1.64	0.83	5.17	13.1%

Supplementary Table 6. EXAFS data fitting results for PBA-0 and PBA-60.

Sample	Path	Coordination number	R (Å)	σ^2 (10^{-3}Å^2)	ΔE_0 (eV)
PBA-0	Ni-N	5.9	2.07	5.3	-3.8
PBA-60	Ni-N	5.5	2.07	5.0	-2.6
PBA-0	Fe-C	6.1	1.9	3.2	-3.8
	Fe-N	6.2	3.4	3.7	-9.4
	Fe-Fe	10.1	2.9	13.3	6.3
PBA-60	Fe-C	5.7	1.9	3.6	-6.0
	Fe-N	5.7	3.3	3.7	-5.3
	Fe-Fe	8.7	2.9	15.6	-0.6

Supplementary References

1. Yin, Y. *et al.* Formation of Hollow Nanocrystals Through the Nanoscale Kirkendall Effect. *Science* **304**, 711-714 (2004).
2. Zhang, W. *et al.* Synthesis of Monocrystalline Nanoframes of Prussian Blue Analogues by Controlled Preferential Etching. *Angew. Chem. Int. Ed.* **55**, 8228-8234 (2016).
3. Ren, W. *et al.* Activation of Sodium Storage Sites in Prussian Blue Analogues via Surface Etching. *Nano Lett.* **17**, 4713-4718 (2017).
4. Nan, H. *et al.* Strong Photoluminescence Enhancement of MoS2 through Defect Engineering and Oxygen Bonding. *ACS Nano* **8**, 5738-5745 (2014).
5. Wu, X., Siu, G., Fu, C. & Ong, H. Photoluminescence and cathodoluminescence studies of stoichiometric and oxygen-deficient ZnO films. *Appl. Phys. Lett.* **78**, 2285-2287 (2001).
6. Lee, H. *et al.* Direct observation of a two-dimensional hole gas at oxide interfaces. *Nat. Mater.* **17**, 231-236 (2018).
7. Nagaraja, P., Kumar, M. S. H., Yathirajan, H. S. & Prakash, J. S. Novel sensitive spectrophotometric method for the trace determination of cyanide in industrial effluent. *Anal. Sci.* **18**, 1027-1030 (2002).
8. Gong, M. *et al.* An Advanced Ni-Fe Layered Double Hydroxide Electrocatalyst for Water Oxidation. *J. Am. Chem. Soc.* **135**, 8452-8455 (2013).
9. You, Y., Yu, X., Yin, Y., Nam, K.-W. & Guo, Y.-G. Sodium iron hexacyanoferrate with high Na content as a Na-rich cathode material for Na-ion batteries. *Nano Res.* **8**, 117-128 (2015).
10. Glatzel, P., Jacquamet, L., Bergmann, U., de Groot, F. M. F. & Cramer, S. P. Site-Selective EXAFS in Mixed-Valence Compounds Using High-Resolution Fluorescence Detection: A Study of Iron in Prussian Blue. *Inorg. Chem.* **41**, 3121-3127 (2002).
11. Su, X. *et al.* Operando Spectroscopic Identification of Active Sites in NiFe Prussian Blue Analogues as Electrocatalysts: Activation of Oxygen Atoms for Oxygen Evolution Reaction. *J. Am. Chem. Soc.* **140**, 11286-11292 (2018).
12. Kang, J.-S. *et al.* Valence and spin states in delafossite AgNiO2 and the frustrated Jahn-Teller system ANiO2 (A= Li, Na). *Phys. Rev. B* **76**, 195122 (2007).
13. Zheng, X. *et al.* Theory-driven design of high-valence metal sites for water oxidation confirmed using in situ soft X-ray absorption. *Nat. Chem.* **10**, 149-154 (2018).
14. Xu, J. *et al.* Elucidation of the surface characteristics and electrochemistry of high-performance LiNiO2. *Chem. Commun.* **52**, 4239-4242 (2016).
15. Chiang, Y.-D. *et al.* Rational Design and Synthesis of Cyano-Bridged Coordination Polymers with Precise Control of Particle Size from 20 to 500 nm. *Eur. J. Inorg. Chem.* **2013**, 3141-3145 (2013).
16. Kulesza, P. J. Solid-state electrochemistry of iron hexacyanoferrate (Prussian Blue type) powders: Evidence for redox transitions in mixed-valence ionically conducting microstructures. *J. Electroanal. Chem.* **289**, 103-116 (1990).
17. McCrory, C. C. L., Jung, S., Peters, J. C. & Jaramillo, T. F. Benchmarking Heterogeneous Electrocatalysts for the Oxygen Evolution Reaction. *J. Am. Chem. Soc.* **135**, 16977-16987 (2013).
18. Soler-Illia, G. J. d. A. A., Jobbágy, M., Regazzoni, A. E. & Blesa, M. A. Synthesis of Nickel Hydroxide by Homogeneous Alkalinization. Precipitation Mechanism. *Chem. Mater.* **11**, 3140-3146 (1999).
19. Cheng, M.-Y. & Hwang, B.-J. Control of uniform nanostructured α-Ni(OH)2 with self-assembly sodium dodecyl sulfate templates. *J. Colloid Interface Sci.* **337**, 265-271 (2009).

20. Song, J. *et al.* Removal of Interstitial H2O in Hexacyanometallates for a Superior Cathode of a Sodium-Ion Battery. *J. Am. Chem. Soc.* **137**, 2658-2664 (2015).
21. Lee, J.-H., Ali, G., Kim, D. H. & Chung, K. Y. Metal‐Organic Framework Cathodes Based on a Vanadium Hexacyanoferrate Prussian Blue Analogue for High‐Performance Aqueous Rechargeable Batteries. *Adv. Energy Mater.* **7**, 1601491 (2016).
22. Zhang, Y., Yang, M., Dou, X.-M., He, H. & Wang, D.-S. Arsenate Adsorption on an Fe−Ce Bimetal Oxide Adsorbent: Role of Surface Properties. *Environ. Sci. Technol.* **39**, 7246-7253 (2005).
23. Tian, X. *et al.* Microwave-assisted non-aqueous homogenous precipitation of nanoball-like mesoporous α-Ni(OH)2 as a precursor for NiOx and its application as a pseudocapacitor. *J. Mater. Chem.* **22**, 8029-8035 (2012).
24. Nayak, S., Mohapatra, L. & Parida, K. Visible light-driven novel g-C3N4/NiFe-LDH composite photocatalyst with enhanced photocatalytic activity towards water oxidation and reduction reaction. *J. Mater. Chem. A* **3**, 18622-18635 (2015).
25. Chapman, K. W., Chupas, P. J. & Kepert, C. J. Compositional Dependence of Negative Thermal Expansion in the Prussian Blue Analogues MIIPtIV(CN)6 (M = Mn, Fe, Co, Ni, Cu, Zn, Cd). *J. Am. Chem. Soc.* **128**, 7009-7014 (2006).
26. Mažeikienė, R., Niaura, G. & Malinauskas, A. Electrochemical redox processes at cobalt hexacyanoferrate modified electrodes: An in situ Raman spectroelectrochemical study. *J. Electroanal. Chem.* **719**, 60-71 (2014).
27. Rao, R., Achary, S., Tyagi, A. & Sakuntala, T. Raman spectroscopic study of high-pressure behavior of Ag3[Co(CN)6]. *Phys. Rev. B* **84**, 054107 (2011).
28. Louie, M. W. & Bell, A. T. An Investigation of Thin-Film Ni−Fe Oxide Catalysts for the Electrochemical Evolution of Oxygen. *J. Am. Chem. Soc.* **135**, 12329-12337 (2013).
29. Liu, X., Zhou, K., Wang, L., Wang, B. & Li, Y. Oxygen Vacancy Clusters Promoting Reducibility and Activity of Ceria Nanorods. *J. Am. Chem. Soc.* **131**, 3140-3141 (2009).
30. Guo, Y. *et al.* Engineering the Electronic State of a Perovskite Electrocatalyst for Synergistically Enhanced Oxygen Evolution Reaction. *Adv. Mater.* **27**, 5989-5994 (2015).
31. Kim, J., Yin, X., Tsao, K.-C., Fang, S. & Yang, H. Ca2Mn2O5 as Oxygen-Deficient Perovskite Electrocatalyst for Oxygen Evolution Reaction. *J. Am. Chem. Soc.* **136**, 14646-14649 (2014).
32. Ling, T. *et al.* Engineering surface atomic structure of single-crystal cobalt (II) oxide nanorods for superior electrocatalysis. *Nat. Commun.* **7**, 12876 (2016).
33. Wang, Y. *et al.* Reduced Mesoporous Co3O4 Nanowires as Efficient Water Oxidation Electrocatalysts and Supercapacitor Electrodes. *Adv. Energy Mater.* **4**, 1400696 (2014).
34. Xu, W. *et al.* Porous cobalt oxide nanoplates enriched with oxygen vacancies for oxygen evolution reaction. *Nano Energy* **43**, 110-116 (2018).
35. Yan, K.-L. *et al.* A facile method for reduced CoFe2O4 nanosheets with rich oxygen vacancies for efficient oxygen evolution reaction. *Int. J. Hydrogen Energy* **42**, 24150-24158 (2017).
36. Zhu, K. *et al.* Perovskites decorated with oxygen vacancies and Fe-Ni alloy nanoparticles as high-efficiency electrocatalysts for the oxygen evolution reaction. *J. Mater. Chem. A* **5**, 19836-19845 (2017).
37. Zhuang, L. *et al.* Ultrathin Iron-Cobalt Oxide Nanosheets with Abundant Oxygen Vacancies for the Oxygen Evolution Reaction. *Adv. Mater.* **29**, 1606793 (2017).
38. Liu, Y. *et al.* Low Overpotential in Vacancy-Rich Ultrathin CoSe2 Nanosheets for Water Oxidation. *J. Am. Chem. Soc.* **136**, 15670-15675 (2014).

39. Zhu, J. *et al.* Co-vacancy-rich Co1–xS nanosheets anchored on rGO for high-efficiency oxygen evolution. *Nano Res.* **10**, 1819-1831 (2017).
40. Zhu, Y. *et al.* Enhancing Electrocatalytic Activity of Perovskite Oxides by Tuning Cation Deficiency for Oxygen Reduction and Evolution Reactions. *Chem. Mater.* **28**, 1691-1697 (2016).
41. Liu, R., Wang, Y., Liu, D., Zou, Y. & Wang, S. Water-Plasma-Enabled Exfoliation of Ultrathin Layered Double Hydroxide Nanosheets with Multivacancies for Water Oxidation. *Adv. Mater.* **29**, 1701546 (2017).
